# The rocker-soled shoes change the kinematics and muscle contractions of the lower extremity during various functional movement

**DOI:** 10.1038/s41598-022-25116-2

**Published:** 2022-11-28

**Authors:** Chao-Yen Chen, You-De Yeh, Ying-Cheng Chen, Pin-Hung Chuang, Hwai-Ting Lin

**Affiliations:** 1 Physical Education Center, Kaohsiung Medical University, Kaohsiung, Taiwan; 2 Department of Sports Medicine, College of Medicine, Kaohsiung Medical University, Kaohsiung, 807 Taiwan; 3 Department of Physical Education, National Kaohsiung Normal University, Kaohsiung, Taiwan; 4 Department of Leisure and Sports Management, Cheng Shiu University, Kaohsiung, Taiwan; 5 Department of Medical Research, Kaohsiung Medical University Hospital, Kaohsiung, Taiwan

**Keywords:** Neuroscience, Physiology, Health care, Risk factors

## Abstract

While rocker-shaped soles have become popular for running shoes, whether or not this type of shoe benefits other functional movements has rarely been discussed. The purpose of this study was to investigate the effect of rocker-soled shoes on lower extremity biomechanics during different exercises. Seventeen healthy university students were recruited. A motion capture analysis system and surface electromyography were used to measure kinematics and muscle activation while walking (10 m), running (10 m), cutting, jumping, and ascending and descending stairs. The results showed that when wearing rocker-soled shoes, greater peak external ankle rotation was present during most exercises. Smaller peak joint angles were observed in hip extension and external rotation when walking, and in ankle dorsiflexion when ascending stairs and jumping. The vastus medialis and vastus lateralis contracted more in most exercises when rocker-soled shoes were worn. However, the biceps femoris and medial gastrocnemius showed less muscle contraction. Wearing rocker-soled shoes during testing movements change the kinematics and muscle contractions of the lower extremity. These findings may provide information for choosing shoes for different exercises or training purposes.

## Introduction

There is a wide range of footwear on the market, and manufacturers frequently claim to have unique designs and technologies for different needs and customers. Rocker-soled shoes are such a design that has been functionally designed for walking and running. Previous studies have shown that these shoes can cause significant changes in lower extremity biomechanics^1–5^. MBT shoes (Masai Barefoot Technology, Switzerland), which were probably the first rocker-soled shoes, were invented by observing the walking patterns of the Masai people. These MBT shoes were constructed with the sole rounded in the anterior–posterior direction. One theory regarding how MBT shoes work is that they transform flat, hard surfaces, into uneven surfaces^6^. The shoes may act as an unstable training device, since this shoe features are thought to specifically activate and strengthening the smaller extrinsic foot muscles while walking or running. By activating these muscles, it was proposed that joint loadings could be reduced since shorter muscle moment arm of the extrinsic muscles compared to the larger muscles of the triceps surae^7^, thus making it easier to walk and run^6^. Moreover, ankle joint with stronger foot extrinsic muscles could more effectively control ankle joint movement to achieve stability^6^.

Previous studies applied MBT M-model (apex was at 54% of the shoe length from the heel)^1^ and modified from commercial shoes (apex was at 60% of the shoe length from the heel, a stiffened rocker profile by a certified orthopedic shoe technician)^4,5^, all of these studies found that rocker-soled shoes reduced ankle plantar flexion moment and power generation^1,4,5^, possibly helping reduce Achilles tendon loading at the start of the push-off phase while running. This helps to prevent or treat Achilles tendinopathy in runners^1,8^. Rocker-soled shoes also aid in muscle recovery after intense exercise. Nakatawa et al.^9^ found that wearing MBT shoes reduced pain and enhanced thigh muscle recovery during post-marathon recovery. Because of the unstable nature of rocker-soled shoes, they can also be used to train the neuromuscular system, which becomes effective after wearing the shoes for longer periods of time. Some studies found that prolonged use of rocker-soled shoes (8–10 weeks) helped participants improve postural control performance and decreased center of pressure displacement while standing^10,11^.

Although rocker-soled shoes may reduce ankle plantarflexion moment and power absorption, one study found that running with rocker-soled shoes may increase knee positive work, extension moment, and extension impulse. These could increase the risk of knee overuse injuries^5^. The arc of the sole’s curve is in the anterior–posterior direction and thus may be more suitable for movements in this direction, such as running and walking. However, some exercises and competitive sports require movements in multiple directions or changes in direction. Whether rocker-soled shoes are suitable for such sports has rarely been discussed in previous studies. Wearing rocker-soled shoes affects muscle activation during exercise, but previous studies have reported inconsistent results^4,12^.

Many studies have reported that wearing rocker-soled shoes can change the kinematics of walking and running in the lower extremities^13–16^, but whether rocker-soled shoes are beneficial or harmful for other functional movements during daily activities or exercise, such as ascending and descending stairs, jumping, or cutting, requires further investigation. Therefore, the aim of this study was to investigate the effect of rocker-soled shoes on lower extremity kinematics and muscle activation during functional movements.

## Results

The results found that all variables were normally distributed. The maximum joint angles in three motion planes (sagittal plane, frontal plane, and transverse plane) of the hip, knee, and ankle joint during each functional task are shown in Tables 1, 2 and 3. Larger ankle external rotation angles were found during the walking, running, ascending and descending stair movements when wearing rocker-soled shoes. Additionally, larger ankle plantar flexion during ascending stair movements was observed when wearing rocker-soled shoes. We also observed that when wearing rocker-soled shoes, there were smaller joint angles during hip external rotation when walking; smaller ankle dorsiflexion angles when ascending stairs; and smaller ankle dorsiflexion angles during both the ascending and descending phases of jumping. Details of each subject’s range of motion of three lower extremity joints in five different activities could be found in Supplementary.

**Table 1:** Extreme ankle joint angles (°) in three motion planes during six functional movements (mean ± standard deviation).

	Plantar flexion (+)/dorsiflexion (−)		Inversion (+)/eversion (−)		External rotation (+)/internal rotation (−)	
	Max	Min	Max	Min	Max	Min
**Walking**						
R	21.8 ± 4.7	− 0.5 ± 4.6	14.4 ± 6.0	8.5 ± 3.0	**11.5 ± 5.7**^**a**^	3.7 ± 6.0
N	23.6 ± 4.4	− 1.4 ± 4.3	14.2 ± 6.3	6.9 ± 3.0	9.1 ± 5.6	1.9 ± 6.4
**Running**						
R	32.2 ± 5.5	− 11.2 ± 2.9	16.7 ± 4.8	6.4 ± 3.2	**17.1 ± 7.7**^**a**^	2.3 ± 5.6
N	32.8 ± 4.8	− 11.8 ± 4.0	16.5 ± 5.2	4.9 ± 3.1	15.3 ± 7.8	1.5 ± 6.3
**Descending stairs**						
R	21.9 ± 8.8	− 24.0 ± 4.5	16.9 ± 3.5	4.9 ± 3.3	**17.5 ± 6.8**^**a**^	4.2 ± 5.5
N	22.4 ± 9.5	− 24.5 ± 4.7	15.7 ± 4.5	3.3 ± 3.3	15.5 ± 6.6	3.0 ± 5.9
**Ascending stairs**						
R	**24.8 ± 4.4**^**a**^	− 6.4 ± 3.4	12.5 ± 4.4	5.5 ± 2.7	**17.2 ± 6.9**^**a**^	1.4 ± 4.7
N	23.1 ± 3.8	** − 9.0 ± 3.0**^**b**^	12.6 ± 3.7	4.1 ± 3.0	15.9 ± 6.9	1.5 ± 5.4
**Jumping (descending)**						
R	31.9 ± 6.8	− 14.5 ± 6.3	18.9 ± 4.6	8.8 ± 8.2	18.5 ± 6.6	1.9 ± 5.2
N	32.5 ± 5.0	** − 19.2 ± 5.3**^**b**^	17.9 ± 4.6	7.3 ± 4.2	19.5 ± 6.9	2.2 ± 4.3
**Jumping (ascending)**						
R	30.4 ± 4.8	− 19.7 ± 6.5	15.5 ± 3.2	5.6 ± 4.3	19.5 ± 6.2	1.9 ± 4.5
N	30.9 ± 4.9	** − 23.9 ± 3.2**^**b**^	14.8 ± 4.2	3.0 ± 5.0	19.6 ± 6.0	0.0 ± 4.3
**Cutting**						
R	37.5 ± 8.0	− 9.7 ± 8.8	22.6 ± 6.6	8.6 ± 4.4	18.5 ± 8.7	− 1.4 ± 7.0
N	37.3 ± 6.6	− 11.2 ± 9.0	20.9 ± 6.5	8.2 ± 3.7	15.5 ± 6.7	− 0.8 ± 5.4

*N* normal shoes, *R* rocker-soled shoes.

^a^Represents an angle for rocker-soled shoes that is significantly larger than for normal shoes (*p* < 0.007).

^b^Represents an angle for normal shoes that is significantly larger than for rocker-soled shoes (*p* < 0.007).

Significant values are in bold.

**Table 2:** Extreme knee joint angles (°) in three motion planes during six functional movements (mean ± standard deviation).

	Flexion		Adduction (+)/abduction (−)		External rotation (+)/internal rotation (−)	
	Max	Min	Max	Min	Max	Min
**Walking**						
R	44.8 ± 7.8	8.8 ± 4.9	5.5 ± 2.6	1.9 ± 2.3	8.7 ± 6.3	0.2 ± 5.3
N	46.2 ± 4.0	6.5 ± 4.2	5.7 ± 2.7	1.8 ± 2.6	10.2 ± 6.9	0.1 ± 5.5
**Running**						
R	44.1 ± 3.8	18.0 ± 3.8	5.8 ± 3.2	1.7 ± 2.4	6.4 ± 7.5	2.1 ± 7.0
N	42.4 ± 5.4	17.1 ± 4.9	6.0 ± 3.3	1.7 ± 2.6	6.8 ± 8.8	1.2 ± 5.9
**Descending stairs**						
R	85.0 ± 6.2	15.6 ± 3.5	8.7 ± 4.5	1.8 ± 3.3	7.9 ± 6.6	5.6 ± 5.7
N	82.5 ± 10.6	14.9 ± 3.8	8.6 ± 5.0	1.9 ± 2.5	7.8 ± 6.1	5.9 ± 6.6
**Ascending stairs**						
R	70.7 ± 6.4	16.5 ± 5.0	10.3 ± 4.9	2.4 ± 1.9	3.8 ± 6.4	4.8 ± 5.3
N	71.6 ± 2.7	15.8 ± 4.7	10.4 ± 4.8	2.3 ± 2.3	4.2 ± 5.7	4.6 ± 5.3
**Jumping (descending)**						
R	64.2 ± 11.4	11.1 ± 6.3	5.8 ± 3.6	1.7 ± 2.4	6.1 ± 5.3	6.4 ± 5.7
N	67.5 ± 11.4	9.6 ± 5.9	6.9 ± 4.3	1.4 ± 3.0	5.5 ± 6.3	7.7 ± 6.5
**Jumping (ascending)**						
R	98.0 ± 11.4	9.6 ± 5.9	8.9 ± 4.6	1.5 ± 3.0	4.9 ± 4.9	11.6 ± 5.6
N	98.8 ± 10.5	11.1 ± 6.3	9.0 ± 4.8	2.4 ± 1.9	4.8 ± 4.9	13.3 ± 7.3
**Cutting**						
R	54.7 ± 9.3	17.0 ± 4.6	7.3 ± 3.5	− 0.7 ± 2.5	6.2 ± 8.3	10.3 ± 7.8
N	53.1 ± 9.9	16.0 ± 3.3	7.6 ± 4.1	− 0.2 ± 3.3	6.8 ± 8.3	10.6 ± 7.2

*N* normal shoes, *R* rocker-soled shoes.

^a^Represents an angle for rocker-soled shoes that is significantly larger than for normal shoes (*p* < 0.007).

^b^Represents an angle for normal shoes that is significantly larger than for rocker-soled shoes (*p* < 0.007).

**Table 3:** Extreme hip joint angles (°) in three motion planes during six functional movements (mean ± standard deviation).

	Flexion (+)/extension (−)		Abduction (+)/adduction (−)		External rotation (+)/internal rotation (−)	
	Max	Min	Max	Min	Max	Min
**Walking**						
R	26.9 ± 8.9	− 12.7 ± 6.6	12.3 ± 7.0	− 1.8 ± 3.9	8.4 ± 8.8	0.1 ± 9.0
N	26.3 ± 8.8	− 14.2 ± 6.2	12.0 ± 6.0	− 2.3 ± 4.5	**9.9 ± 7.2**^**b**^	1.0 ± 7.7
**Running**						
R	27.7 ± 7.0	− 5.9 ± 7.2	8.4 ± 7.1	− 4.0 ± 4.3	8.0 ± 7.4	− 1.2 ± 7.0
N	27.5 ± 7.9	− 6.6 ± 5.1	8.3 ± 8.0	− 2.8 ± 3.8	8.5 ± 6.4	− 0.9 ± 8.4
**Descending stairs**						
R	22.4 ± 7.2	8.5 ± 6.7	10.2 ± 4.4	− 0.6 ± 4.8	8.3 ± 9.2	− 5.4 ± 8.6
N	21.5 ± 6.4	5.9 ± 8.6	10.9 ± 6.4	0.2 ± 6.0	9.6 ± 9.9	− 4.5 ± 8.3
**Ascending stairs**						
R	57.9 ± 14.1	0.6 ± 6.8	12.8 ± 13.8	− 1.6 ± 3.5	9.3 ± 8.5	− 6.4 ± 8.0
N	57.8 ± 13.4	− 0.2 ± 6.3	12.9 ± 12.0	− 0.6 ± 4.1	9.4 ± 9.0	− 6.8 ± 7.3
**Jumping (descending)**						
	35.8 ± 14.9	2.5 ± 8.5	16.1 ± 7.4	8.1 ± 3.2	13.2 ± 6.9	− 4.2 ± 9.0
N	33.5 ± 14.4	4.4 ± 6.4	15.3 ± 6.0	7.9 ± 4.5	12.1 ± 7.9	− 3.2 ± 7.9
**Jumping (ascending)**						
R	75.5 ± 13.3	4.5 ± 6.3	15.2 ± 6.0	7.6 ± 2.8	13.9 ± 7.6	1.0 ± 6.0
N	75.5 ± 14.3	5.7 ± 6.8	16.4 ± 6.4	7.7 ± 3.2	12.4 ± 7.2	− 0.1 ± 7.0
**Cutting**						
R	37.4 ± 7.4	− 6.9 ± 7.9	13.9 ± 6.5	1.7 ± 4.6	8.9 ± 11.3	− 10.5 ± 11.4
N	37.1 ± 10.0	− 6.0 ± 7.1	14.7 ± 7.4	2.9 ± 4.8	9.7 ± 10.3	− 8.9 ± 10.1

*N* normal shoes, *R* rocker-soled shoes.

^a^Represents an angle for rocker-soled shoes significantly larger than for normal shoes (*p* < 0.007).

^b^Represents an angle for normal shoes significantly larger than for rocker-soled shoes (*p* < 0.007).

Significant values are in bold.

The mean activation of six muscles during each functional task is shown in Table 4. When wearing rocker-soled shoes, the vastus medialis showed greater muscle activation while walking, and ascending and descending stairs, and vastus lateralis showed greater activation when ascending stairs and cutting. However, when wearing rocker-soled shoes less muscle contraction was observed in the biceps femoris during walking, and cutting movements; in the medial gastrocnemius while ascending stairs; and in the vastus medialis while jumping (ascending). Averaged activation details of six muscles per subject in six different activities are in Supplementary.

**Table 4:** Mean activation (% MVC) of six muscles during each functional task.

	VM	TA	VL	PF	BF	GA
**Walking**						
R	**8.5 ± 3.5**^**a**^	13.4 ± 4.7	11.9 ± 3.7	23.7 ± 7.4	9.6 ± 3.2	24.9 ± 9.7
N	7.0 ± 2.6	15.3 ± 5.9	11.0 ± 3.7	22.9 ± 6.1	**12.5 ± 5.1**^**b**^	29.1 ± 16.6
**Running**						
R	34.2 ± 11.1	28.8 ± 13.1	28.8 ± 8.6	52.3 ± 18.9	29.7 ± 10.8	43.2 ± 22.8
N	33.8 ± 10.0	32.8 ± 9.7	25.0 ± 6.2	53.2 ± 19.5	30.6 ± 11.7	51.3 ± 25.7
**Descending stairs**						
R	**21.8 ± 5.4**^**a**^	12.1 ± 5.3	17.3 ± 4.5	16.4 ± 5.3	6.3 ± 2.3	18.5 ± 7.4
N	18.6 ± 5.4	14.0 ± 6.3	16.2 ± 4.7	16.9 ± 5.2	6.9 ± 2.4	15.7 ± 5.5
**Ascending stairs**						
R	**29.2 ± 11.1**^**a**^	10.9 ± 3.5	**25.6 ± 5.6**^**a**^	27.5 ± 7.2	13.8 ± 4.3	24.4 ± 6.1
N	24.2 ± 7.1	12.0 ± 3.6	21.9 ± 4.5	30.0 ± 11.9	13.4 ± 4.0	**28.7 ± 7.9**^**b**^
**Jumping (descending)**						
R	27.2 ± 6.5	17.1 ± 7.4	25.3 ± 6.5	34.8 ± 10.6	12.1 ± 4.9	37.5 ± 9.4
N	27.8 ± 7.6	18.3 ± 7.0	26.7 ± 8.1	32.8 ± 12.3	13.0 ± 6.3	37.9 ± 8.1
**Jumping (ascending)**						
R	32.8 ± 6.9	28.9 ± 9.0	29.6 ± 9.8	21.4 ± 8.7	8.6 ± 3.1	15.7 ± 5.4
N	**40.8 ± 8.2**^**b**^	35.7 ± 11.6	32.5 ± 9.4	28.4 ± 14.5	11.6 ± 6.3	20.2 ± 6.7
**Cutting**						
R	44.0 ± 14.7	25.9 ± 9.9	**35.8 ± 11.5**^**a**^	55.1 ± 17.6	29.3 ± 8.9	49.1 ± 17.7
N	36.7 ± 5.7	23.1 ± 7.7	31.0 ± 10.9	60.9 ± 17.5	**36.4 ± 14.7**^**b**^	54.2 ± 16.1

*BF* biceps femoris, *GA* gastrocnemius, *MVC* maximum isometric voluntary contraction, *N* normal shoes, *PF* peroneus longus, *R* rocker-soled shoes, *TA* tibial anterior, *VL* vastus lateralis, *VM* vastus medialis.

^a^Muscle activation in rocker-soled shoes was significantly larger than in normal shoes (*p* < 0.007).

^b^Muscle activation in normal shoes was significantly larger than in rocker-soled shoes (*p* < 0.007).

Significant values are in bold.

## Discussion

Most prior studies found that wearing rocker-soled shoes affected joint movement in the sagittal and frontal planes, but not in the transverse plane^14^. However, this study found that wearing rocker-soled shoes significantly increased the external rotation of the ankle when walking and running which was not consistent with a previous study showing that rocker-soled shoes increased ankle dorsiflexion in the sagittal plane^1^. This inconsistency may result from the rocker-soled shoes feel relatively unstable when worn^4,16^, our participants’ unfamiliarity with wearing rocker-soled shoes, which could have led them to increase externally rotated their ankles to increase the base of support in medial–lateral direction to maintain body stability. This study also showed that activation of the vastus medialis was significantly increased, and activation of the biceps femoris was significantly decreased, when wearing rocker-soled shoes while walking, in agreement with the findings of a previous study^12^. The greater quadriceps contractions was to stabilize the body, while the lower bicep femoris activation may come from the smaller hip extension. We further demonstrated that the six muscles did not show any significant differences in activation between the two types of shoes when the participants were running, although, the gastrocnemius trended towards lower activation in the rocker-soled shoe group. Donoghue^20^ reported that Achilles tendinopathy is due to larger ankle joint dorsiflexion and plantarflexion moments. A recent study also found that rocker-soled shoes could decrease the ankle’s plantarflexion moment during the propulsion phase and decrease activation of the gastrocnemius muscle. Therefore, rocker-soled shoes may help to relieve pain in patients with Achilles tendinopathy.

When wearing rocker-soled shoes while ascending stairs, the ankle dorsiflexion angle was significantly decreased, whereas ankle plantarflexion, and external rotation, angles were significantly increased. Ankle external rotation was also observed to increase during the ascending and descending stair movements, to stabilize the body. The increase in ankle plantarflexion and decrease in dorsiflexion may correlate with the rocker shape of the shoes^1,4^. When pushing off to ascend the stairs, the rocker shape may have helped the participants to roll their feet upwards with less effort. In this situation, activation of the vastus medialis and vastus lateralis increased, while gastrocnemius activation was decreased. Shinno^17^ reported that the stability of the body is strongly dependent on the quadriceps while descending stairs. When wearing rocker-soled shoes, the first contact area is the apex of the shoe. Because of the rocker shape, the contact surface then becomes smaller, such that the quadriceps needs to contract more to stabilize the body. Moreover, the peroneus longus did not show any significant difference between the two types of shoes during the ascending and descending stair movements, indicating that the shape of the sole may not influence the medio-lateral inversion of the ankle.

The ankle dorsiflexion angle significantly decreased during the ascending phase of jumping when wearing rocker-soled shoes. The dorsiflexion of the ankle joint in the squat position at the beginning of the jump is more stable when wearing normal shoes. However, when wearing rocker-soled shoes, the anterior–posterior rocker shape makes earlier roll-off^1,4,5^. This may result in the ankle dorsiflexion angle being significantly smaller with rocker-soled shoes than with normal shoes during counter-movement jumps. In addition, the activation of the vastus medialis, biceps femoris, and gastrocnemius was significantly lower with rocker-soled shoes than with normal shoes. This may also be related to the lower ankle dorsiflexion angle.

The ankle dorsiflexion angle was significantly reduced in the descending phase of jumping. More stability is required when landing, which demands ankle dorsiflexion and knee flexion to absorb the shock^18^. The rocker shape of the sole could potentially help to absorb the energy when landing. We therefore hypothesized that the participants would display reduced ankle joint dorsiflexion when landing after jumps. However, in the muscle activation analyses, we did not find any significant difference between the two types of shoes. In this study, jump height was not measured. Whether wearing rocker-soled shoes results in better performance under the same muscle activation conditions could be confirmed in the study.

Ankle inversion was almost reached significant increased (*0.007 < p < 0.05*) during cutting movements when wearing rocker-soled shoes. Ankle inversion and eversion are especially important during cutting. Rocker-soled shoes have an anterior–posterior rocker design, thus when the participants needed to change direction, a higher force was needed. The muscle activation analyses showed that the activation of the vastus medialis and vastus lateralis was significantly increased and the activation of the biceps femoris was significantly decreased while cutting. Participants were asked to move towards a target at an oblique angle of 45° and then to immediately rotate the ankle 90° to continue running at right angles. When the foot strikes the ground with a large of force, the quadriceps needs to contract more to stabilize the knee joint. When wearing rocker-soled shoes, there is more instability in this scenario, and thus the participants may have used greater quadriceps contractions to maintain posture.

The rocker profile and material properties of the shoes may affect the kinematics and muscle contractions during different functional exercises. In this study, the rocker profile of the testing shoes was similar to the MBT shoes having rounded heel as well as a toe rocker^4,5^, however, some studies tested the shoes only have a toe rocker^1^. Moreover, the tested shoes were with a stiffened rocker profile in most of the studies, the material properties of the outsoles in our testing shoes from heel to the toe is the same. The weight of the tested shoes also different between previous studies (700–900 g) and this study (290 g).

Previous studies found gender differences in kinetics and balance during functional movement. Therefore, one limitation of this study was that all the participants were male. The measures taken in this study focused on joint kinematics, but with further analysis of kinetics and foot pressure data, the discussion could be extended to joint loading and options for reducing exercise injuries when wearing rocker-soled shoes. Many of the kinematic changes when wearing rocker-soled shoes observed in this study may correlate to the muscles around the hip. However, the muscles around the hip were not investigated during functional movement testing, representing another limitation. Rocker-soled shoe mechanics could be explored in more detail in further studies.

Wearing rocker-soled shoes significantly increased the activation of the vastus medialis and vastus lateralis muscles and decreased gastrocnemius activation, especially while ascending stairs. Wearing rocker-soled shoes also reduced muscle activation when ascending during jumping. Greater external rotation of the ankle, plantarflexion, inversion angles, and hip abduction, but less hip extension, external rotation, and ankle dorsiflexion occurred when wearing rocker-soled shoes during functional movement testing. This information may help people to choose appropriate shoes for different exercises.

## Methods

### Participants

Seventeen healthy male students were recruited for this study, with an average age of 22.0 ± 1.6 years, an average height of 176.4 ± 3.4 cm, and an average weight of 68.2 ± 5.6 kg. Sample power calculation was based on knee joint range of motion for repeated measures, large effect size (= 0.8), power of 80%, and alpha error of 5%. We informed the students of the purpose and contents of this study received written consent before participating. The study protocol was approved by the Kaohsiung Medical University Hospital Institutional Review Board and all the testing procedures have been performed in accordance with the Declaration of Helsinki. The inclusion criteria were collegiate students, 20–30 years old, without any musculoskeletal injuries in the 6 months prior to the study, and no prior experience in wearing rocker-soled shoes. The exclusion criteria were the presence of cardiovascular or respiratory diseases, joint instability, or laxity in the lower extremities, or balance disorders occurring in the 6 months before the study.

### Test shoes

**Figure 1:**
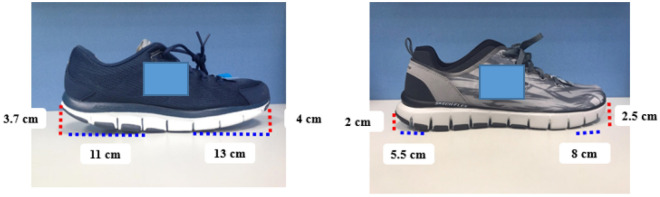
The dimensions of the two shoe types tested in this study. The left panel shows the rocker-soled shoe, and the right panel displays the normal shoe.

We selected one type of rocker-soled shoe and one type of normal-soled shoe of the same brand (Fig. 1). As previous studies reported that an abrupt change from a lightweight to a heavy shoe can induce immediate alterations in muscular activity^19^, we selected two shoes of approximately the same weight. The rocker-soled shoes weighed 293.8 g and the normal shoes weighed 262.0 g. The two shoe types had similar soles in terms of material properties, stiffness, and hardness. The dimensions of the two types of shoes (size US: 9, shoe length is 27 cm) are shown in Fig. 1.

### Study procedures

This study was a crossover study. All participants were informed of the procedures and of the benefits and risks of the study and signed informed consent forms before beginning the tests. To quantify the participants’ hip, knee, and ankle joint kinematics during functional movement, a Qualisys™ motion capture system (Qualisys AB, Sweden) with six infrared cameras was used at a 200 Hz sampling rate. Twenty-one infrared reflective markers were attached to selected anatomical positions based on Helen Hays marker sets. The positions were: the anterior superior iliac spine, the lateral aspect of the thigh and shank, the medial and lateral femoral condyles, the medial and lateral malleolus, the heel, the head of the first and fifth metatarsals (bilaterally), and the sacrum. The TELEMYO 2400 electromyography (EMG) system (Noraxon, USA) was used for muscle activation detection at a sampling 1000 Hz while six functional movements were performed. The bipolar electrodes of the electromyography system were placed directly onto skin that had been prepared with an alcohol wipe to avoid signal interference. The application of surface electrodes were positioned over the vastus medialis, vastus lateralis, tibialis anterior, peroneus longus, biceps femoris, and medial gastrocnemius and followed the recommendations of SENIAM^21^. Participants performed maximal voluntary contractions (MVC) tests by applying strong manual resistance with specific positions for muscle activations presentation. Before the tests, the participants practiced the test movements several times to familiarize themselves with the experimental procedures using rocker-soled or normal-soled shoes. The static standing neutral position was collected twice in order to define the initial relative orientation between the proximal and distal segments. Following the collection of static data, six functional movements including walking 10 m, running 10 m, ascending and descending stairs, cutting, and counter-movement jumps were performed. The order of shoes tested, and test movements were randomized and 3 days elapsed between testing the two shoe types. All six functional movements were repeated three times with a 30 s break between trails and a three-minute rest between each functional movement to prevent muscle fatigue during tests. The muscles activation data (EMG) were collected synchronized with the kinematics data by motion captured system.

### Data analysis

Qualisys Track Manager software (Qualisys AB, Sweden) was used to track marker positions during functional movements in three dimensions. The kinematics data were analyzed with a self-coded program using MATLAB 7.0 (The MathWorks, Inc., USA). For each trial, three hip, knee, and ankle joint angles (the flexion/extension angle, the abduction/adduction angle, and the axial rotational angle) were calculated using Euler’s method with a y–x′–z″ rotational sequence. The EMG signals were analyzed using MyoResearch software (Noraxon). Electrode artifacts were removed from the raw EMG data with a bandpass filter between 20 and 450 Hz (Butterworth, 4th order), and the data were rectified and smoothed. Muscle activation presentation was normalized to the muscle’s maximum isometric voluntary contraction (% MVC). The stance phases of walking, running, cutting, and ascending and descending stairs were analyzed for the parameters presented in this study. Jumping was divided into two phases: the start of the jump (ascending), and landing (descending). The start and end points of the stance phase were judged by the ground reaction force at a threshold of 10 N and measured with a force plate (Type 9286, Kistler, Switzerland).

### Statistical analysis

All data analyses were performed using SPSS statistical software, version 19 (SPSS Inc., Chicago, Illinois, USA). The data were tested for normality using the Shapiro–Wilk test. ANOVA tests for repeated measures was used to examine muscle activation differences and hip, knee, and ankle joint angles when wearing the two different types of shoes during five functional movement testing. To protect against Type I error, Bonferroni’s correction was used. Thus, the level of statistical significance was set at *p* = 0.007 for each comparisons.

### Ethical approval

This study was approved by the Institute of Review Board, Kaohsiung Medical University Chung-Ho Memorial Hospital. The IRB number is KMUHIRB-E(I)-20160177.

## Supplementary Information

Supplementary Information 1.

Supplementary Information 2.

Supplementary Information 3.

Supplementary Information 4.

## Data Availability

The data sets used and/or analyzed within the current study are available from the corresponding author on reasonable request.
