# Supplementary material for: The rocker-soled shoes change the kinematics and muscle contractions of the lower extremity during various functional movement
Source: Sci Rep. 2022 Nov 28;12:20523. doi: 10.1038/s41598-022-25116-2 (PMC9705322; doi:10.1038/s41598-022-25116-2)
Supplement: Supplementary file 1 — Supplementary Information 1. [file 41598_2022_25116_MOESM1_ESM.pdf]

Plantar flexion

	Cutting		Decending Stair		Ascending stairs		Jumping (ascending)		Jumping (descending)		Running		Walking	
Subject	Normal	Rocker	Normal	Rocker	Normal	Rocker	Normal	Rocker	Normal	Rocker	Normal	Rocker	Normal	Rocker
1	35.51	31.84	27.87	30.73	22.99	23.06	39.51	29.73	23.11	32.13	33.90	32.70	19.67	26.00
2	38.12	43.03	27.29	26.19	23.05	22.33	26.11	29.92	28.21	31.35	35.23	37.81	33.08	25.09
3	27.53	20.55	7.44	7.68	22.40	25.42	34.11	30.63	35.70	35.91	32.40	26.99	19.91	15.14
4	36.28	35.07	14.90	18.40	24.78	24.69	26.30	35.18	34.67	40.61	31.70	32.65	19.70	20.98
5	48.62	45.85	27.83	30.42	24.34	28.03	36.11	28.43	38.96	34.35	32.10	31.44	24.20	26.00
6	35.60	36.23	31.91	33.15	27.62	25.72	28.00	30.57	36.36	20.13	32.27	38.71	22.78	20.88
7	43.90	44.05	9.07	18.01	28.12	30.54	32.83	35.57	31.25	30.06	37.09	38.18	27.36	29.82
8	39.51	38.12	25.34	25.88	24.01	24.66	31.04	36.80	33.23	30.04	31.19	29.39	21.88	17.48
9	34.34	49.94	8.73	8.28	22.38	25.02	30.40	29.31	34.85	39.03	26.96	29.57	21.61	17.63
10	39.44	38.76	28.38	29.27	25.45	27.62	34.97	33.91	35.45	39.77	36.15	34.59	27.52	22.98
11	45.98	42.47	36.58	33.31	29.62	35.28	34.36	28.41	38.14	31.26	45.12	40.42	30.20	31.83
12	43.93	38.99	28.05	26.59	17.50	21.56	28.82	37.07	32.38	27.16	31.51	27.61	28.45	23.22
13	30.38	36.21	21.70	17.13	25.45	22.99	36.92	21.93	26.05	18.34	24.25	19.73	22.97	17.91
14	35.12	33.26	28.30	22.03	17.81	19.76	21.03	33.37	24.30	27.50	33.05	26.04	23.25	19.30
15	41.92	43.80	28.24	18.84	19.45	20.33	31.15	30.70	29.04	43.99	25.85	32.89	22.13	17.21
16	35.33	20.37	6.07	5.61	21.17	28.31	29.66	24.63	39.44	30.76	32.29	38.05	16.22	19.67
17	22.63	39.81	23.91	20.75	16.39	16.97	23.73	20.40	32.19	30.04	36.15	30.77	19.45	19.41
AVE	37.30	37.55	22.45	21.90	23.09	24.84	30.89	30.39	32.55	31.91	32.78	32.21	23.55	21.80
SD	6.64	7.95	9.47	8.77	3.76	4.36	4.92	4.82	4.97	6.77	4.76	5.46	4.42	4.67

Dorsiflexion(+)/Plantar flexion(-)

	Cutting		Decending Stair		Ascending stairs		Jumping (ascending)		Jumping (descending)		Running		Walking	
Subject	Normal	Rocker	Normal	Rocker	Normal	Rocker	Normal	Rocker	Normal	Rocker	Normal	Rocker	Normal	Rocker
1	12.45	29.82	31.07	28.04	11.99	5.18	22.44	21.23	23.95	17.40	14.30	12.81	2.84	-2.67
2	27.10	23.08	20.29	16.56	8.60	10.11	25.21	24.23	21.26	23.82	14.40	13.03	1.31	2.31
3	15.83	15.30	24.17	30.08	7.40	5.05	27.05	24.55	25.55	20.45	11.27	10.09	4.18	4.92
4	9.50	11.74	26.47	25.45	13.24	11.63	26.20	21.98	26.07	6.48	19.02	18.18	8.90	6.52
5	22.76	15.54	28.86	28.08	4.40	3.78	20.95	23.92	11.86	10.36	14.04	14.33	-0.62	-2.84
6	-3.18	-1.94	25.87	22.76	7.13	1.19	27.12	16.78	17.38	11.49	9.27	6.34	5.45	0.13
7	6.15	6.95	19.65	21.41	3.65	2.11	21.77	8.99	14.90	17.34	11.73	8.88	-2.33	-4.88
8	0.58	4.23	15.36	18.35	8.63	5.00	19.74	19.30	14.21	13.59	1.26	7.33	-0.58	0.44
9	14.60	10.79	21.81	20.41	9.93	5.86	21.31	23.60	20.97	13.80	8.03	8.36	3.55	4.63
10	19.04	8.70	25.04	25.05	9.32	9.13	26.10	15.61	22.07	7.64	11.11	10.47	-2.98	-2.82
11	1.12	1.63	16.40	16.37	4.97	1.46	19.04	22.93	9.93	11.66	8.27	10.31	-5.58	-8.21
12	6.36	7.34	26.95	26.42	10.23	11.13	22.56	25.94	15.87	22.82	14.04	11.57	-4.37	-4.78
13	25.35	18.47	24.75	23.84	11.75	6.75	29.68	23.93	23.71	23.08	14.12	10.28	2.38	2.12
14	5.43	6.99	27.67	23.65	13.04	10.62	24.76	18.56	22.27	15.42	14.86	13.63	9.08	9.39
15	2.00	-3.62	27.13	27.96	12.00	8.23	20.07	21.21	16.62	16.74	15.53	12.61	3.88	4.82
16	13.79	7.79	32.31	31.70	10.53	8.90	28.37	21.37	26.62	13.91	11.98	12.57	3.45	2.29
17	12.31	1.38	22.16	22.60	6.28	3.60	23.22	7.80	13.66	8.90	8.19	10.70	-3.13	-1.50
AVE	11.25	9.66	24.47	24.04	9.01	6.45	23.86	20.11	19.23	14.99	11.85	11.26	1.50	0.58
SD	8.95	8.78	4.71	4.49	3.00	3.44	3.22	5.23	5.28	5.37	4.04	2.85	4.34	4.67

Inversion(+)(Max)/Eversion(-)

	Cutting		Decending Stair		Ascending stairs		Jumping (ascending)		Jumping (descending)		Running		Walking	
Subject	Normal	Rocker	Normal	Rocker	Normal	Rocker	Normal	Rocker	Normal	Rocker	Normal	Rocker	Normal	Rocker
1	29.06	29.53	23.37	20.58	17.99	13.27	19.10	18.54	26.28	19.30	25.36	17.84	23.62	18.31
2	27.98	28.26	12.41	14.79	14.16	13.06	16.97	16.19	17.74	22.43	18.62	20.81	16.57	17.81
3	18.51	24.03	9.56	18.24	11.78	16.03	9.04	14.97	12.66	18.53	14.41	19.56	13.92	18.69
4	24.13	26.90	13.71	15.24	12.95	11.62	13.16	17.97	20.89	26.72	14.15	15.00	15.25	13.99
5	22.44	27.33	17.07	22.74	15.24	18.65	14.04	5.38	17.53	10.61	17.24	19.72	19.67	18.87
6	10.25	9.98	10.93	9.60	6.14	4.39	6.65	16.78	10.37	11.49	7.24	4.36	5.07	5.20
7	6.15	6.95	19.65	21.41	3.65	2.11	21.77	13.90	14.90	22.27	11.73	8.88	-2.33	-4.88
8	20.21	23.77	15.34	15.99	9.96	10.79	12.07	16.08	18.00	20.96	18.00	19.28	15.69	17.96
9	23.82	30.51	16.94	16.82	14.99	13.32	16.40	18.24	22.51	28.00	19.19	15.29	17.16	15.96
10	27.34	27.96	21.07	19.47	16.55	18.07	21.11	14.62	26.84	17.20	23.20	21.54	19.22	17.68
11	24.75	24.35	20.66	19.37	13.39	10.08	15.57	11.94	18.36	16.51	19.20	17.94	16.10	12.91
12	17.61	16.54	15.01	14.11	13.98	11.46	11.24	19.52	15.00	17.98	17.69	16.34	16.53	16.44
13	24.81	25.29	14.32	17.71	14.98	12.67	18.32	16.85	15.79	19.18	15.18	14.83	14.43	13.23
14	27.59	22.63	20.49	17.72	15.93	17.42	17.71	17.11	20.49	18.97	24.60	25.28	20.29	16.79
15	17.62	20.44	16.26	18.12	12.73	15.71	14.15	16.20	18.28	19.48	14.26	16.85	13.72	16.82
16	20.16	21.38	12.18	14.63	10.66	13.58	14.48	15.67	16.13	16.71	13.66	16.96	9.43	15.34
17	13.13	18.67	7.14	10.93	9.40	10.57	10.40	14.30	11.88	14.20	6.90	13.56	7.09	12.96
AVE	20.92	22.62	15.65	16.91	12.62	12.52	14.83	15.54	17.86	18.85	16.51	16.71	14.20	14.36
SD	6.49	6.56	4.46	3.49	3.73	4.38	4.17	3.23	4.56	4.56	5.23	4.83	6.32	5.97

Inversion(+)(Min)/Eversion(-)

	Cutting		Decending Stair		Ascending stairs		Jumping (ascending)		Jumping (descending)		Running		Walking	
Subject	Normal	Rocker	Normal	Rocker	Normal	Rocker	Normal	Rocker	Normal	Rocker	Normal	Rocker	Normal	Rocker
1	12.74	10.06	0.25	-0.05	3.13	3.48	-2.07	10.68	2.19	14.80	3.03	3.65	12.26	8.65
2	11.22	13.80	4.58	8.19	7.05	5.72	8.95	0.23	14.38	5.83	6.42	8.21	6.43	10.25
3	5.17	13.95	-1.22	3.33	0.97	7.66	-4.79	5.31	0.32	9.50	3.65	7.26	4.92	9.87
4	4.38	12.55	3.96	4.45	1.86	4.65	3.07	6.95	3.54	11.66	2.25	5.01	6.41	7.67
5	13.44	12.46	2.37	4.98	3.16	6.80	2.98	-2.04	5.71	3.20	4.02	7.48	9.06	11.07
6	0.86	0.47	0.11	-0.10	-3.11	-2.93	-6.24	-3.54	3.15	-20.13	-3.32	-2.03	0.19	-1.31
7	9.07	12.23	1.97	5.87	6.40	5.45	-2.23	8.14	4.35	10.12	2.26	4.12	6.56	7.35
8	9.27	3.44	0.74	1.94	0.26	3.98	0.37	7.73	4.62	13.36	4.62	2.73	7.02	8.41
9	10.62	1.57	11.65	10.21	6.25	5.39	7.36	5.95	9.57	12.96	9.25	8.46	10.13	9.61
10	9.70	9.14	3.40	1.94	7.95	7.51	7.17	6.69	9.32	9.81	7.84	8.49	8.87	9.27
11	8.21	9.16	6.84	8.03	5.27	4.27	7.21	4.09	8.30	9.03	7.94	8.56	7.85	7.33
12	3.13	1.99	5.35	4.32	5.43	5.10	5.84	1.77	10.65	6.38	5.80	5.82	5.94	6.23
13	9.07	12.23	1.97	5.87	6.40	5.45	-2.23	8.14	4.35	10.12	2.26	4.12	6.56	7.35
14	13.53	9.20	5.01	4.02	6.71	7.78	9.16	10.93	14.48	16.55	8.90	6.95	9.39	9.22
15	9.43	9.30	7.42	11.27	5.46	10.34	8.74	10.76	12.47	14.46	7.31	11.42	8.25	12.21
16	6.43	5.93	1.84	6.77	2.40	6.61	4.27	8.67	8.09	12.14	5.80	9.01	6.10	11.31
17	3.77	8.32	-0.25	2.48	4.06	5.60	3.44	4.87	8.45	9.85	5.37	9.96	1.13	10.18
AVE	8.24	8.58	3.29	4.91	4.10	5.46	3.00	5.61	7.29	8.80	4.91	6.42	6.89	8.51
SD	3.74	4.40	3.30	3.27	2.92	2.74	5.02	4.32	4.22	8.19	3.14	3.24	2.97	3.01

External rotation(Max)

	Cutting		Decending Stair		Ascending stairs		Jumping (ascending)		Jumping (descending)		Running		Walking	
Subject	Normal	Rocker	Normal	Rocker	Normal	Rocker	Normal	Rocker	Normal	Rocker	Normal	Rocker	Normal	Rocker
1	12.02	36.92	28.82	29.77	29.54	31.13	30.90	8.79	31.55	8.13	32.73	32.17	20.17	24.08
2	14.80	12.83	3.52	4.02	3.94	5.94	10.68	26.28	10.51	25.58	6.46	6.04	-0.99	-0.06
3	14.78	24.26	15.89	20.51	12.79	18.41	19.18	20.11	18.63	21.72	14.54	19.30	9.25	14.19
4	21.14	18.78	14.43	18.09	15.06	16.54	22.01	25.32	22.37	19.51	14.68	18.44	11.05	13.35
5	7.86	23.62	14.38	19.05	15.68	19.42	21.18	13.15	19.03	10.17	15.03	17.13	9.09	12.63
6	30.31	7.24	11.11	11.29	10.92	9.58	13.73	31.73	12.30	32.91	6.98	7.10	7.89	6.83
7	12.23	31.93	27.08	29.92	28.70	30.12	27.61	18.26	31.56	17.58	29.02	30.75	13.69	20.31
8	10.74	14.57	14.25	16.61	14.12	15.41	14.29	17.98	14.87	15.10	11.51	14.87	10.60	11.91
9	26.76	14.32	9.12	9.56	13.54	13.31	17.63	29.58	17.08	28.29	8.24	10.16	5.03	6.65
10	22.36	25.84	25.32	27.00	23.66	24.99	29.99	22.86	31.56	21.05	24.24	26.10	14.17	16.07
11	9.06	23.26	18.64	18.32	20.50	16.94	23.55	15.64	20.79	12.92	23.27	23.90	15.13	14.17
12	22.44	10.36	11.24	13.18	11.23	13.40	15.32	23.83	13.55	24.85	9.70	10.87	4.99	6.51
13	15.68	26.58	18.23	20.03	19.33	21.52	22.86	18.55	22.89	19.54	16.35	19.35	10.62	13.93
14	12.19	15.15	13.71	15.33	18.50	17.47	21.27	15.19	22.11	16.72	16.75	15.99	11.44	11.12
15	9.20	12.59	15.57	17.31	15.74	16.71	15.86	13.03	17.17	12.65	13.57	15.49	8.31	9.55
16	8.89	8.42	12.03	13.82	8.75	9.91	15.30	16.71	15.97	13.92	7.43	9.29	7.79	8.60
17	12.55	8.60	10.44	14.19	8.64	11.48	12.00	15.32	10.01	14.52	9.23	13.88	-2.64	6.07
AVE	15.47	18.55	15.52	17.53	15.92	17.19	19.61	19.55	19.53	18.54	15.28	17.11	9.15	11.52
SD	6.72	8.75	6.58	6.83	6.90	6.85	6.08	6.23	6.93	6.65	7.84	7.67	5.57	5.74

External rotation(+)/Internal rotation (-)(Min)

	Cutting		Decending Stair		Ascending stairs		Jumping (ascending)		Jumping (descending)		Running		Walking	
Subject	Normal	Rocker	Normal	Rocker	Normal	Rocker	Normal	Rocker	Normal	Rocker	Normal	Rocker	Normal	Rocker
1	11.10	14.34	12.65	13.10	12.04	8.76	2.26	-8.92	2.26	-5.02	15.13	9.95	14.46	14.67
2	-10.91	-7.21	-8.54	-7.20	-8.49	-8.40	-10.63	4.09	-5.27	7.76	-11.34	-10.67	-12.35	-11.52
3	0.34	7.97	3.78	9.85	-1.20	4.69	-3.21	-0.08	1.83	0.61	1.00	6.38	1.16	3.77
4	1.87	-2.19	3.35	4.30	2.72	1.24	-1.31	4.71	0.35	1.28	0.15	0.44	4.49	3.99
5	-5.82	-4.63	4.03	3.89	0.91	0.25	1.11	0.09	3.12	-0.50	-0.20	1.01	1.83	-0.83
6	-0.17	-1.79	-1.96	-2.32	-2.76	-1.46	-1.75	9.65	0.45	14.49	-2.59	-0.06	-0.19	-0.84
7	4.14	3.03	15.11	12.60	10.64	10.33	3.67	7.62	10.22	1.75	13.05	12.07	9.98	10.31
8	1.31	-1.36	7.31	7.21	6.51	5.10	9.65	-0.85	3.48	-1.93	6.61	4.71	7.99	8.33
9	-3.38	-14.23	0.94	2.14	-2.53	-2.83	-0.67	6.99	-0.34	5.96	-2.23	-2.26	-2.82	-0.28
10	-4.07	-6.35	8.64	10.30	4.22	0.63	4.96	4.64	6.27	3.27	6.10	5.37	7.47	9.89
11	1.92	1.54	2.90	3.10	6.21	1.86	0.76	-0.11	3.87	-0.36	3.95	6.56	6.47	3.04
12	-5.96	-5.86	-1.54	-0.65	-2.66	-1.90	-0.22	3.90	0.66	9.28	-2.04	-0.81	-2.89	-1.15
13	7.58	8.65	5.53	8.59	1.67	6.84	0.37	2.18	7.67	4.01	4.49	9.08	1.30	9.16
14	0.77	-7.29	-0.66	0.58	3.79	0.65	2.36	-1.53	8.37	1.42	-2.07	-1.25	0.74	3.05
15	-4.02	-5.40	-0.18	3.38	1.97	2.74	-2.86	-2.98	1.46	-6.05	-0.48	1.29	-0.52	3.90
16	-4.56	1.16	2.86	2.56	-2.85	-3.44	-3.21	2.43	-3.88	-1.18	-1.57	-2.63	3.02	4.68
17	-3.45	-4.71	-3.87	0.68	-3.90	-1.30	-1.41	0.52	-3.43	-2.40	-2.18	0.54	-7.23	2.14
AVE	-0.78	-1.43	2.96	4.24	1.55	1.40	-0.01	1.90	2.18	1.90	1.52	2.34	1.94	3.67
SD	5.38	7.01	5.87	5.46	5.38	4.72	4.28	4.45	4.29	5.23	6.29	5.59	6.44	5.97
