# Supplementary material for: The rocker-soled shoes change the kinematics and muscle contractions of the lower extremity during various functional movement
Source: Sci Rep. 2022 Nov 28;12:20523. doi: 10.1038/s41598-022-25116-2 (PMC9705322; doi:10.1038/s41598-022-25116-2)
Supplement: Supplementary file 2 — Supplementary Information 2. [file 41598_2022_25116_MOESM2_ESM.pdf]

VMO(%MVC)

	Cutting		Decending Stair		Ascending stairs		Jumping (ascending)		Jumping (descending)		Running		Walking	
Subject	Rocker	Normal	Rocker	Normal	Rocker	Normal	Rocker	Normal	Rocker	Normal	Rocker	Normal	Rocker	Normal
1	62.10	34.60	21.50	19.80	30.30	28.10	36.70	46.60	28.70	29.70	39.70	47.40	8.50	7.30
2	63.60	76.20	19.00	12.50	37.80	28.60	40.40	38.70	32.70	25.10	18.20	19.50	3.70	5.50
3	28.90	41.60	18.30	14.10	11.70	11.90	23.70	46.90	23.80	19.70	33.80	35.90	9.30	6.90
4	24.60	20.60	15.60	14.40	25.00	24.50	33.70	33.70	23.30	18.90	29.20	33.60	4.90	4.10
5	52.80	42.60	19.00	16.00	30.40	24.60	33.40	35.40	19.70	25.50	56.90	54.80	7.10	7.40
6	42.10	40.10	24.10	23.60	43.20	30.60	41.40	37.80	27.40	28.60	39.60	40.10	5.30	5.20
7	53.30	51.40	24.40	12.30	33.00	26.70	36.50	41.60	44.30	38.60	44.10	42.50	8.90	10.60
8	31.30	20.10	26.70	28.50	24.90	20.70	48.00	58.50	28.90	42.60	24.00	23.30	9.40	8.20
9	40.00	31.30	14.80	11.90	22.30	19.10	35.20	36.10	25.80	32.10	34.70	34.10	3.20	2.00
10	43.30	28.80	11.50	17.50	24.80	19.50	28.50	26.60	23.00	24.00	31.70	23.40	12.90	12.50
11	29.50	23.10	21.40	16.80	30.10	30.20	33.80	40.80	23.00	19.00	45.40	31.90	5.60	4.80
12	44.60	22.40	33.90	26.70	26.10	25.60	25.60	43.80	19.20	18.80	13.70	17.50	9.70	5.90
13	42.20	39.30	22.60	17.10	18.30	14.80	31.50	27.20	29.30	31.10	33.90	38.40	17.00	10.90
14	22.60	26.50	22.40	22.30	15.80	17.50	25.80	45.70	30.10	32.60	48.90	40.30	9.20	7.80
15	79.30	57.30	31.60	27.20	59.40	42.20	23.10	49.30	17.90	20.30	25.30	24.80	9.90	6.20
16	48.50	39.50	25.30	20.50	35.50	25.60	35.20	48.50	35.36	25.80	25.90	38.50	12.50	5.90
17	38.90	28.90	18.90	15.60	28.50	20.50	25.30	36.80	29.80	39.50	35.90	29.30	7.50	8.30
AVE	43.98	36.72	21.82	18.64	29.24	24.16	32.81	40.82	27.19	27.76	34.17	33.84	8.51	7.03
SD	14.73	5.71	5.38	5.38	11.06	7.09	6.89	8.17	6.54	7.58	11.12	10.08	3.52	2.61

Tibial anterior(%MVC)

	Cutting		Decending Stair		Ascending stairs		Jumping (ascending)		Jumping (descending)		Running		Walking	
Subject	Rocker	Normal	Rocker	Normal	Rocker	Normal	Rocker	Normal	Rocker	Normal	Rocker	Normal	Rocker	Normal
1	19.00	22.90	9.00	16.00	9.40	8.80	28.90	48.90	32.20	29.90	26.60	34.00	17.80	23.30
2	20.00	20.30	5.20	4.00	7.20	6.80	11.70	12.70	16.60	13.50	23.70	28.20	12.90	14.40
3	37.70	23.70	19.80	21.60	7.90	12.90	36.00	30.30	23.20	12.90	25.10	26.10	10.80	12.30
4	17.30	15.20	13.30	15.30	11.50	14.10	27.30	15.60	21.90	26.10	11.00	14.60	12.80	12.70
5	15.90	14.80	10.50	4.90	6.20	5.00	37.40	33.70	12.30	11.40	16.30	21.20	6.20	6.70
6	16.00	12.40	8.70	9.60	13.40	7.30	40.80	49.50	11.30	11.80	67.60	52.60	10.20	9.50
7	32.20	18.00	22.80	16.50	17.20	13.40	20.40	16.70	13.60	20.10	22.00	39.90	8.20	8.50
8	18.60	18.40	7.70	8.20	11.50	17.90	15.10	42.50	16.00	13.60	31.30	28.80	11.40	18.00
9	21.90	26.40	8.70	6.60	6.60	9.90	18.50	24.00	11.40	28.10	17.60	25.10	11.90	11.70
10	29.60	22.50	21.30	23.50	9.40	11.20	38.70	44.10	17.40	27.70	26.20	25.70	13.60	10.00
11	22.00	16.80	10.10	13.10	9.90	10.00	25.40	34.40	3.10	4.20	26.70	38.50	11.60	27.00
12	23.10	28.90	7.60	21.80	8.10	12.40	19.70	20.10	12.20	15.10	30.40	44.00	15.40	16.80
13	38.00	27.10	11.60	20.10	18.10	15.90	31.20	35.60	22.50	22.60	20.60	25.60	14.70	18.20
14	41.80	43.70	8.90	15.30	12.90	16.70	22.70	24.50	17.80	17.00	40.00	32.00	25.90	21.10
15	47.30	33.50	17.00	20.90	15.00	13.60	21.30	31.10	31.20	16.10	46.90	41.90	20.90	21.80
16	18.50	25.80	9.50	12.80	11.80	13.70	20.90	35.90	15.60	21.20	26.90	39.30	11.30	8.90
17	20.50	22.70	15.90	8.40	9.80	13.50	25.90	44.20	12.30	19.80	30.80	40.50	11.50	18.60
AVE	25.85	23.12	12.21	14.04	10.94	11.95	28.87	35.74	17.09	18.30	28.81	32.82	13.36	15.26
SD	9.94	7.68	5.26	6.28	3.52	3.58	8.98	11.60	7.35	7.04	13.09	9.67	4.69	5.91

VLO(%MVC)

	Cutting		Decending Stair		Ascending stairs		Jumping (ascending)		Jumping (descending)		Running		Walking	
Subject	Rocker	Normal	Rocker	Normal	Rocker	Normal	Rocker	Normal	Rocker	Normal	Rocker	Normal	Rocker	Normal
1	31.70	24.30	15.70	13.30	18.20	17.40	28.20	38.10	23.40	22.70	31.10	27.10	8.80	9.20
2	25.20	29.80	14.90	13.50	24.70	15.80	17.20	16.60	14.50	9.60	24.50	11.60	20.80	7.90
3	27.60	24.20	12.90	11.00	22.70	22.40	26.20	43.30	28.30	32.90	21.20	27.30	9.40	9.60
4	43.80	49.90	15.00	12.10	35.70	32.40	28.10	28.50	23.50	21.90	38.20	24.80	10.70	11.70
5	40.30	35.00	18.40	20.60	30.90	23.50	47.30	48.60	25.00	29.50	25.20	21.80	16.00	15.40
6	71.20	61.50	26.70	15.00	31.00	28.40	30.30	28.60	21.70	22.40	43.80	34.90	12.60	13.70
7	34.40	32.70	20.60	22.00	29.10	24.50	35.50	29.70	34.70	35.10	28.10	31.20	8.10	6.80
8	32.00	26.60	12.60	11.40	20.10	20.00	39.40	46.00	26.20	32.70	16.10	19.50	10.70	16.80
9	42.90	34.20	12.80	16.50	27.70	22.20	20.40	21.50	24.40	26.30	34.80	24.90	7.10	14.10
10	22.40	13.20	18.20	13.90	14.90	14.80	36.00	33.40	30.20	28.50	29.20	17.20	8.40	6.60
11	26.10	20.00	14.30	11.20	21.40	19.30	38.80	31.40	27.10	20.90	11.60	16.80	8.30	8.10
12	40.00	29.00	24.90	23.40	29.30	24.70	16.40	19.10	19.40	23.60	40.10	32.20	14.20	13.80
13	30.00	31.10	15.80	16.90	25.50	25.80	17.40	27.40	42.80	47.20	34.30	29.00	14.30	9.60
14	29.60	29.70	10.60	10.80	18.90	19.10	46.50	40.60	17.30	18.90	34.90	25.60	15.10	13.70
15	27.90	29.00	19.50	18.90	26.30	19.20	20.80	23.60	22.90	23.30	20.30	30.00	9.90	3.50
16	38.50	25.80	22.50	18.70	28.90	21.50	25.90	35.70	23.50	27.50	30.50	22.50	15.30	10.50
17	45.30	30.20	18.90	25.40	30.50	20.50	29.20	40.30	25.30	30.20	25.90	28.70	12.50	15.30
AVE	35.82	30.95	17.31	16.15	25.64	21.85	29.62	32.49	25.31	26.66	28.81	25.01	11.89	10.96
SD	11.52	10.91	4.51	4.66	5.57	4.48	9.76	9.44	6.50	8.12	8.59	6.16	3.67	3.73

Peroneus(%MVC)

	Cutting		Decending Stair		Ascending stairs		Jumping (ascending)		Jumping (descending)		Running		Walking	
Subject	Rocker	Normal	Rocker	Normal	Rocker	Normal	Rocker	Normal	Rocker	Normal	Rocker	Normal	Rocker	Normal
1	41.40	52.60	12.20	20.30	23.80	19.50	21.20	28.30	40.30	32.10	42.20	68.90	29.40	25.50
2	68.50	81.10	19.40	17.80	44.60	18.60	22.80	57.90	29.50	24.10	52.50	29.90	31.70	25.00
3	43.30	73.70	11.40	12.50	29.90	33.60	16.90	15.10	40.40	47.80	45.70	47.40	25.40	26.80
4	44.00	42.80	6.80	10.80	14.40	22.30	17.90	16.90	37.70	40.40	31.50	22.50	11.80	12.90
5	30.00	38.60	13.00	12.30	24.70	22.70	15.50	18.70	27.20	21.90	26.30	41.10	17.10	15.00
6	38.00	39.20	30.20	25.70	25.00	20.20	50.00	53.30	17.80	19.70	31.90	36.60	16.50	14.80
7	57.70	44.50	13.40	9.70	21.10	20.60	16.20	18.40	66.30	64.20	91.80	92.30	21.00	20.00
8	79.20	84.00	23.60	17.70	33.40	59.50	29.20	28.60	35.70	35.50	70.10	89.60	43.40	33.00
9	78.80	66.30	17.00	23.30	23.10	21.80	26.10	23.60	42.20	34.00	30.00	48.50	23.90	22.60
10	58.80	52.60	16.60	18.30	36.00	45.80	18.10	17.70	28.80	27.80	77.20	52.50	29.00	28.70
11	98.40	90.10	19.20	11.80	37.80	45.70	21.90	52.50	37.00	52.50	60.60	76.40	24.30	27.30
12	54.70	32.70	14.40	16.30	26.90	31.60	23.70	12.30	32.70	26.10	66.00	37.50	15.80	20.20
13	44.90	78.80	12.10	21.00	26.50	31.60	13.30	13.70	30.00	34.30	42.10	40.40	18.40	19.80
14	51.40	66.00	15.20	11.10	28.60	27.60	22.60	41.20	36.60	27.80	56.40	60.20	24.40	26.40
15	43.50	65.80	17.30	25.60	20.40	17.60	10.60	25.80	20.70	21.70	34.60	57.70	26.00	33.10
16	57.80	66.80	15.80	18.90	23.50	31.50	19.80	30.50	38.80	25.90	60.80	51.20	25.30	15.80
17	45.70	59.80	20.50	14.30	27.90	40.20	18.50	28.30	29.50	22.50	68.60	52.50	20.30	22.60
AVE	55.06	60.91	16.36	16.91	27.51	30.02	21.43	28.40	34.78	32.84	52.25	53.25	23.75	22.91
SD	17.57	17.48	5.34	5.16	7.25	11.87	8.69	14.50	10.63	12.26	18.86	19.50	7.38	6.13

Biceps femoris(%MVC)

	Cutting		Decending Stair		Ascending stairs		Jumping (ascending)		Jumping (descending)		Running		Walking	
Subject	Normal	Rocker	Normal	Rocker	Normal	Rocker	Normal	Rocker	Normal	Rocker	Normal	Rocker	Normal	Rocker
1	42.20	64.10	5.90	6.50	25.20	20.00	18.10	26.70	12.40	15.50	50.20	62.00	10.30	15.00
2	26.10	37.10	3.30	7.70	16.50	24.10	7.90	18.20	10.50	14.30	16.10	28.60	12.00	11.40
3	24.60	15.20	8.00	6.60	11.40	11.00	7.70	6.90	9.90	9.90	32.10	26.90	9.10	5.40
4	23.10	36.00	10.70	7.90	11.10	10.50	6.80	7.90	10.20	9.10	26.30	26.20	14.80	15.30
5	47.10	45.10	9.50	9.40	13.00	10.70	9.70	9.60	12.10	18.00	51.20	46.80	16.40	25.40
6	36.60	50.10	7.30	6.50	10.60	13.70	5.70	4.90	24.00	17.10	39.80	42.00	7.80	10.80
7	29.10	29.40	5.30	12.70	12.70	13.20	12.60	15.50	15.40	17.50	36.90	28.20	9.00	17.00
8	21.10	39.40	3.90	4.30	11.20	12.30	7.20	8.70	4.30	5.10	30.70	43.00	4.80	7.40
9	21.20	18.10	5.30	4.90	6.90	11.00	8.40	7.60	8.80	8.70	22.40	22.20	4.00	5.70
10	32.60	34.00	4.70	5.80	13.00	10.00	7.90	9.50	5.00	4.30	15.70	18.60	9.80	10.90
11	23.60	32.60	7.60	6.40	13.00	14.30	9.80	23.20	17.50	29.70	23.30	18.50	8.90	15.10
12	30.60	27.40	6.40	8.90	15.70	14.20	5.80	5.50	8.40	6.60	31.00	35.10	12.10	12.30
13	18.50	21.00	5.30	4.90	10.40	10.70	5.60	7.40	17.00	14.20	25.00	22.80	10.70	17.80
14	17.60	17.70	2.20	2.10	11.50	7.90	6.30	7.80	8.70	7.50	24.10	24.10	5.70	7.10
15	42.90	63.10	5.10	6.30	17.00	13.10	7.60	9.50	12.20	15.20	14.40	20.40	8.50	8.20
16	35.40	48.90	8.60	6.70	15.30	13.50	8.70	15.30	13.20	16.80	30.20	28.40	10.50	15.10
17	25.80	38.90	7.80	9.80	20.30	17.50	10.50	13.20	15.90	11.20	35.10	25.80	8.50	13.20
AVE	29.30	36.36	6.29	6.91	13.81	13.39	8.61	11.61	12.09	12.98	29.68	30.56	9.58	12.54
SD	8.92	14.66	2.26	2.42	4.28	3.98	3.07	6.25	4.87	6.25	10.75	11.70	3.20	5.12

Gastrocnemius(%MVC)

	Cutting		Decending Stair		Ascending stairs		Jumping (ascending)		Jumping (descending)		Running		Walking	
Subject	Normal	Rocker	Normal	Rocker	Normal	Rocker	Normal	Rocker	Normal	Rocker	Normal	Rocker	Normal	Rocker
1	50.60	45.60	10.50	7.70	18.60	16.00	16.70	33.50	30.20	29.00	34.60	32.30	22.80	20.10
2	15.20	31.20	12.20	26.10	29.80	43.80	18.10	23.50	40.10	51.40	12.70	20.80	8.50	11.30
3	71.90	79.40	12.60	17.40	7.70	12.80	7.70	28.10	26.20	29.80	57.10	40.50	14.80	15.90
4	50.70	48.90	34.50	19.50	28.00	39.00	17.80	15.40	30.40	48.00	36.80	47.70	25.20	22.20
5	70.60	64.70	27.60	21.40	29.60	31.10	18.60	23.60	55.60	49.30	110.00	128.00	52.40	53.80
6	49.70	70.30	17.30	15.80	30.30	32.10	29.60	25.60	49.30	43.50	50.90	48.20	21.40	45.10
7	48.30	37.00	15.10	12.10	20.40	18.80	20.20	26.30	39.60	35.60	32.20	47.50	27.70	24.00
8	43.50	42.60	18.70	17.00	21.50	26.20	16.50	19.80	30.60	35.70	19.90	36.00	19.00	19.00
9	55.80	65.70	11.80	7.60	19.20	27.00	9.70	14.40	24.10	31.50	29.50	23.00	24.60	33.80
10	48.60	41.80	19.20	19.60	28.00	32.40	21.30	19.80	34.10	46.60	42.20	51.00	30.00	20.80
11	79.00	77.30	31.00	11.50	27.20	32.00	15.70	16.70	24.10	23.30	59.40	31.60	27.40	42.80
12	64.30	51.40	24.00	6.50	28.40	36.70	14.60	16.90	38.20	41.30	20.80	61.80	31.50	36.60
13	56.20	60.00	19.30	20.60	21.00	23.40	12.00	6.60	49.50	28.90	62.60	70.70	14.80	18.60
14	48.70	80.40	13.10	12.10	32.00	30.00	7.20	9.60	46.00	39.00	57.40	86.90	21.00	38.50
15	19.50	38.70	7.70	15.50	21.40	26.20	12.20	19.60	36.60	38.70	24.10	50.00	21.70	31.20
16	25.80	48.50	16.80	21.50	22.80	29.30	13.50	19.80	38.30	32.80	38.90	44.50	35.90	30.50
17	35.80	38.70	22.50	15.80	28.80	31.50	15.90	23.80	45.30	39.20	45.90	50.80	25.90	30.50
AVE	49.07	54.25	18.46	15.75	24.39	28.72	15.72	20.18	37.54	37.86	43.24	51.25	24.98	29.10
SD	17.65	16.06	7.45	5.54	6.15	7.93	5.42	6.70	9.38	8.11	22.84	25.68	9.72	11.61
